# Characterization and Performance of *Priestia aryabhattai* for Nitrogen Assimilation of Soymilk-Processing Wastewater

**DOI:** 10.3390/biology15181625

**Published:** 2026-09-15

**Authors:** Qianqian Chen, Wentao Yu, Yushu Zhang

**Affiliations:** 1 Fujian Key Laboratory of Plant Nutrition and Fertilizer, Institute of Resources, Environment and Soil Fertilizer, Fujian Academy of Agricultural Sciences, Fuzhou 350003, China; chenqianqian@faas.cn; 2 Fujian Key Laboratory for Technology Research of Inspection and Quarantine, Technology Center of Fuzhou Customs District, Fuzhou 350001, China

**Keywords:** biological nitrogen assimilation, nitrogen metabolism genes, *Priestia*, soymilk-processing wastewater

## Abstract

Soy products are among the most common sources of protein, and large volumes of wastewater are produced from legume processing. In this study, the *Priestia aryabhattai* FM5 strain, isolated from soil, was found to be capable of nitrogen assimilation. The NH_4_^+^ and NO_3_^−^ removal characteristics of *P. aryabhattai* FM5 were investigated to better understand its removal capability. The optimal conditions for NH_4_^+^ removal were pH 8.0, temperature—25 °C, and C/N ratio—20, with sodium succinate as the carbon source. Under the conditions of pH 7.0, temperature—35 °C, and C/N ratio—10, with sodium acetate as the carbon source, strain FM5 reached the highest NO_3_^−^ removal efficiency. The genome of FM5 revealed genes involved in assimilatory nitrate reduction and glutamate metabolism, such as *nasA*, *nirA*, *nrt*, *glnA*, *gltB/D*, and *gdhA*. When cultured in wastewater from soymilk processing with activated carbon for 72 h (h), the NH_4_^+^ concentration and chemical oxygen demand (COD) decreased from 29.5 to 4.8 mg·L^−1^ and 2058.3 mg·L^−1^ to 295.3 mg·L^−1^, respectively. This study provides deeper insights into the NH_4_^+^ and NO_3_^−^ removal-capable bacterial strain FM5 and improves the application of *Priestia* in wastewater.

## Simple Summary

The discharge of ammonium-rich wastewater from legume processing represents a significant environmental concern. In this study, we isolated *Priestia aryabhattai* FM5 from soil and demonstrated its high efficiency in removing both NH_4_^+^ and NO_3_^−^. Whole-genome sequencing identified the presence of key genes involved in nitrogen assimilation. In soymilk-processing wastewater supplemented with activated carbon, treatment with FM5 significantly reduced both ammonium concentration and chemical oxygen demand within 72 h. Our findings highlight FM5 as a promising, cost-effective candidate for sustainable bioremediation of legume-processing wastewater.

## 1. Introduction

Soy products are one of the most important protein sources in the human diet, and large volumes of wastewater are produced during soy processing worldwide [1,2]. Approximately 39 billion tons of soybean-processing wastewater is produced annually in China [3,4]. Soy-processing wastewater has high concentrations of organic materials, and direct disposal leads to significant pollution [5,6,7]. Moreover, water with ammonia nitrogen concentrations exceeding 5 mg·L^−1^ can be highly detrimental to the survival of aquaculture animals and may also pose a threat to human health [7]. Biological nitrogen removal (BNR) methods have been widely recognized for their efficiency in eliminating nitrogen, absence of secondary contamination, and simple operation [8,9,10,11]. These microorganisms are capable of removing nitrogen, especially NH_4_^+^ and NO_3_^−^, through nitrogen metabolism [12,13].

The food industry generates large volumes of wastewater containing high concentrations of organic matter, nitrogen, and phosphorus [5,14]. Numerous bacterial species have been identified for their nitrogen-removal capabilities in water [15,16]. Among them, *Bacillus*, *Pseudomonas*, *Rhodococcus* and *Acinetobacter* have demonstrated high nitrogen-removal efficiencies [17,18,19,20]. Notably, *Bacillus* species have garnered significant attention due to their ability to perform both nitrification and denitrification in a single reactor, thereby reducing the footprint and operational costs of treatment facilities. In addition, *Bacillus* spp. also exhibit multiple advantages in wastewater treatment, such as fast growth rates, high nitrogen removal efficiency, extensive degradation of organic pollutants, and strong environmental adaptability [12]. Many *Bacillus* spp. can be utilized in biological treatment processes to degrade organic pollutants and remove nutrients [21,22]. *B. subtilis* sp. N4, which has been successfully applied in wastewater treatment systems, achieved a nitrite removal efficiency of up to 99% at pH 6.4 and 30 °C [23]. Its ability to thrive in diverse environmental conditions and degrade a wide range of substrates makes it suitable for treating various types of food industry wastewater [24,25]. Additionally, some *Bacillus* spp., such as *B. simplex* H-b, have shown resistance to low temperatures, making them suitable for treating wastewater under low-temperature conditions [26]. *Priestia*, a genus that was renamed from the genus Bacillus based on conserved signature indels in protein sequences, has also been found to have nitrogen assimilation capabilities [27]. *P. aryabhattai* was employed to effectively produce struvite from secondary-treated municipal wastewater, demonstrating a promising strategy for simultaneous phosphorus recovery and wastewater treatment [28]. However, the NH_4_^+^ and NO_3_^−^ removal characteristics and applications of *Priestia* spp. in waste treatment remain less explored compared to those of *Bacillus* spp.

The current study aims to investigate the NH_4_^+^ and NO_3_^−^ removal ability of the *P. aryabhattai* FM5 strain, isolated from soil, and evaluate the impact of culturing conditions on its performance, with the aim of utilizing it for the treatment of soy-processing wastewater. The primary objective of this study is to gain a better understanding of the NH_4_^+^ and NO_3_^−^ removal characteristics of strain FM5 and its potential application in wastewater treatment. In light of these considerations, the culturing conditions were optimized to enhance NH_4_^+^ and NO_3_^−^ removal efficiency. The genome of strain FM5 was evaluated using genomic sequencing to illustrate its pathway associated with nitrogen metabolism. This comprehensive approach not only provides insights into the NH_4_^+^ and NO_3_^−^ removal mechanisms of *P. aryabhattai* FM5 but also highlights its potential as an effective microbial resource for application in wastewater treatment.

## 2. Materials and Methods

### 2.1. Isolation and Culture of Strain FM5

Strains were isolated from the rhizosphere soil of *Lactuca sativa* var. *ramosa* Hort. treated with composting organic fertilizer. The bacteria were first selected using Luria–Bertani (LB) broth medium at 30 °C for 48 h. LB medium contains (g·L^−1^) yeast extract, 5.0 g; peptone, 10.0 g; NaCl, 10.0 g. The single colony was then transformed to NH_4_^+^-N- and NO_3_^−^-N-removal medium. The NH_4_^+^-N-removal medium contains (1 L) C_4_H_4_Na_2_O_4_, 4.1 g; (NH_4_)_2_SO_4_, 0.5 g; Vickers salt solution, 50 mL. The NO_3_^−^-N-removal medium contains (g·L^−1^) KNO_3_, 0.72; KH_2_PO_4_, 1.0; MgSO_4_, 1.0; C_4_H_4_Na_2_O_4_, 2.8. The Vickers salt solution comprises the following components (g·L^−1^): KH_2_PO_4_, 5.0; MgSO_4_·7H_2_O, 2.5; NaCl, 2.8; FeSO_4_·7H_2_O, 0.05; MnSO_4_·4H_2_O, 0.05 [29]. The solid medium was prepared by adding 15 g of agar to 1 L of liquid medium. For the detection of NH_4_^+^- and NO_3_^−^- removal efficiency, ammonia and nitrate contents were determined at 0, 24, 48 and 72 h. Bacterial growth was monitored by measuring the optical density at 600 nm (OD_600_) using a spectrophotometer (SpectraMax M2e, Molecular Devices, San Jose, CA, USA). After centrifugation, the culture supernatant was extracted with a 2 mol·L^−1^ KCl solution, and the resulting extract was subsequently analyzed for NH_4_^+^-N and NO_3_^−^-N concentrations by a Continuous Flow Analytical System (Skalar San^++^ System, Breda, The Netherlands) according to the manufacturer’s protocol.

### 2.2. Identification of Strain FM5

To identify the isolated strain, microscopy and the 16S ribosomal ribonucleic acid (rRNA) gene were analyzed, as previously described [30]. Briefly, genomic DNA was obtained using a DNA extraction kit (Generay, Shanghai, China). The 16S rRNA was amplified using universal primers 27F and 1492R. The partial 16S rRNA gene sequences were sequenced by Biosun Biotechnology Co., Ltd. (Fuzhou City, China). The sequences were edited with BioEdit version 7.2.5 and then analyzed using the Basic Local Alignment Search Tool (BLAST) program in NCBI (https://blast.ncbi.nlm.nih.gov/Blast.cgi, 20 May 2024). A phylogenetic tree was constructed using the neighbor-joining method in the MEGA 5.0 software with 1000 bootstrap replicates. Simultaneously, the complete genome of FM5 was also sequenced. Further, based on 31 housekeeping genes (e.g., *dnaG*, *ropB* and *rplA*), and phylogenetic tree was constructed using the neighbor-joining method in the MEGA 6.0 software.

### 2.3. Determination of NH_4_^+^ and NO_3_^−^ Removal Capacities

The strains that grew well on both heterotrophic nitrification medium (HNM) and aerobic denitrification medium (ADM) were selected using an isolation assay. The colony of the selected strain was inoculated into LB broth and cultured at 30 °C for 24 h at 180 rpm. Then, 1% (*v*/*v*) culture solution was inoculated into the HNM and ADM. The ammonia and nitrate contents were determined at 0, 24, 48 and 72 h. Further, culture conditions, including temperature, pH, carbon sources, C/N ratios and salt concentrations, were assessed. The FM5 strain was precultured in LB medium to OD_600_ = 1.0; then, the precultured suspension was inoculated at a 1% proportion into HNM and ADM. The temperatures were set as 20°, 25°, 30°, 35° and 40 °C. Growth at different pH values (5, 6, 7, 8 and 9) was also assessed. C/N ratios (5, 10, 15, 20 and 25) were investigated at 25 °C, pH 7.0. In the carbon source experiments (C/N = 15), sodium citrate (C_6_H_5_Na_3_O_7_), sodium acetate (C_2_H_3_NaO_2_), sodium succinate (C_4_H_4_Na_2_O_4_), glycerin, and glucose were selected as the sole carbon sources of the. NH_4_^+^ and NO_3_^−^ removal capacities in different salt concentrations (0, 2.5 and 5%) were further analyzed. The NH_4_^+^ and NO_3_^−^ removal efficiencies were calculated by Equation (1):

(1) $$\mathrm{Removal}\;\mathrm{efficiency}\;\left(\%\right)=\frac{C_{0}-C_{1}}{C_{0}}\times 100\;C_{0}\;=\;\mathrm{initial}\;{{\mathrm{NH}}_{4}}^{+}/{{\mathrm{NO}}_{3}}^{-}\;\mathrm{concentration}\;(\mathrm{mg}\cdot L^{-1})\;C_{1}\;=\;\mathrm{final}\;{{\mathrm{NH}}_{4}}^{+}/{{\mathrm{NO}}_{3}}^{-}\;\mathrm{concentration}\;(\mathrm{mg}\cdot L^{-1})$$

### 2.4. Complete Genome Sequencing and Annotation

The FM5 strain was inoculated into 200 mL of LB liquid medium and cultured at 37 °C for approximately 12 h at 180 rpm. The cell biomass was harvested after 5 min of centrifugation at 12,000× *g*. Genomic DNA of FM5 was extracted using the Genomic DNA Extraction Kit (Generay, Shanghai, China) according to the manufacturer’s protocol. Purified genomic DNA was quantified by a TBS-380 fluorometer (Turner BioSystems Inc., Sunnyvale, CA, USA). High-quality DNA was used for further research. The draft genome sequence analysis of the FM5 strain was carried out using the Hiseq X Ten sequencing platform (MajorBio Co., Shanghai, China). Briefly, DNA samples were sheared into 400–500 bp fragments. Illumina sequencing libraries were prepared from the sheared fragments using the NEXTflex™ Rapid DNA-Seq Kit (Bioo Scientific Corporation, Austin, TX, USA). The prepared libraries were then used for paired-end Illumina sequencing (2 × 150 bp) on an Illumina HiSeq X Ten sequencing platform (Illumina, San Diego, CA, USA). The data generated from the Illumina platform were used for bioinformatics analysis. All of the analyses were performed using the free online platform of the Majorbio Cloud Platform (www.majorbio.com) from Shanghai Majorbio Bio-pharm Technology Co., Ltd. (Shanghai, China). The detailed procedures are as follows. Raw reads obtained after sequencing were filtered using the fastp software (version 0.19.6), followed by assembly with SOPA de novo version 2.04 [31]. The final assembled genome had a length of 4,022,950 bp and was submitted to GenBank with the accession number PRJNA1522652. Glimmer was used for coding sequence (CDS) prediction, tRNA-scan-SE was used for tRNA prediction, and Barrnap was used for rRNA prediction. The predicted CDSs were annotated using the Non-Redundant Protein Database (NR) and the Kyoto Encyclopedia of Genes and Genomes (KEGG) database using sequence alignment tools. Briefly, each set of query proteins was aligned with the databases, and annotations of best-matched subjects (e-value < 10^−5^) were obtained for gene annotation.

### 2.5. NH_4_^+^-N Removal in Soymilk Wastewater by Strain FM5

To identify the NH_4_^+^ and NO_3_^−^ removal abilities of strain FM5, 4 treatments were conducted: (1) a blank control, (2) treatment with bacterial strain FM5, (3) treatment with activated carbon, and (4) treatment with both bacterial strain FM5 and activated carbon. After incubation, the chemical oxygen demand (COD) and concentrations of NH_4_^+^-N in each treatment were measured. Wastewater from soymilk production was collected by Yangchen Food Co., Ltd. in Sanming, Fujian Province, China. Before the treatment of wastewater, strain FM5 was cultured in LB liquid at 25 °C for 24 h and then centrifuged (5000 rpm, 10 min). The cell pellet was washed twice with sterile water. Then, the liquid was added to the wastewater at 5.0% (*v*/*v*). In the treatment setup with strain FM5 and biochar, 10 g of activated carbon (Yuanli Active Carbon Co., Ltd., Nanping, China) was added to 1 L of wastewater. The wastewater was cultured in 4 treatments at 25 °C for 72 h. After 24, 48 and 72 h of treatment, the NH_4_^+^-N concentrations and COD were detected by a Continuous Flow Analytical System (Skalar San^++^ System, Breda, The Netherlands) according to the manufacturer’s protocol [32]. All experiments were performed with 3 replicates. Statistical significance among multiple groups was assessed by one-way ANOVA with Tukey’s HSD post hoc test.

## 3. Results

### 3.1. Isolation and Identification of Strain FM5

**Figure 1:**
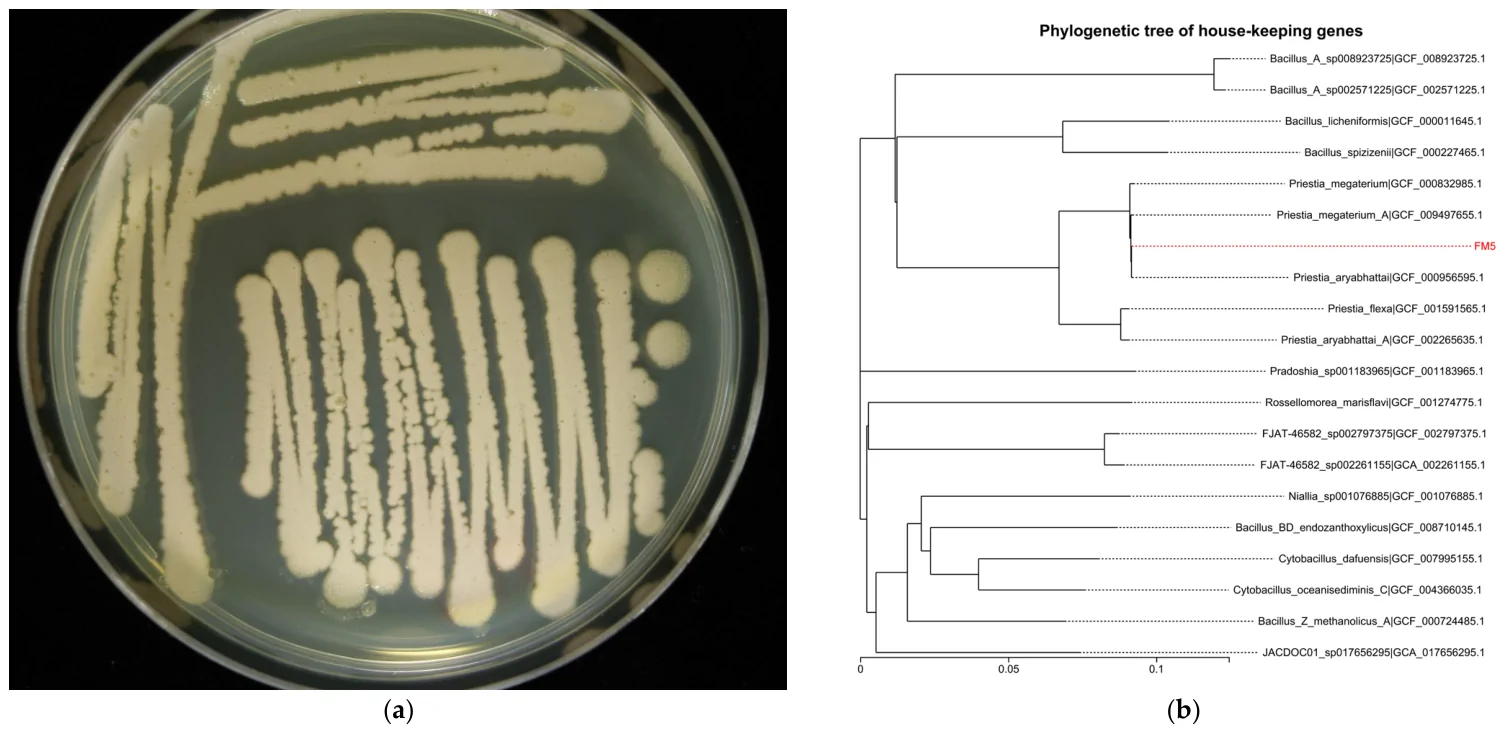
The colonies of strain FM5 (**a**) and phylogenetic tree (**b**) based on housekeeping genes.

The colony characteristics and cell morphology of strain FM5 were consistent with those of *Bacillus*. Colonies grown on LB agar for 48 h were white, dry and flat. Growth of strain FM5 was observed in the temperature range of 25 °C to 50 °C (optimum 30 °C), with a pH range of 5.0 to 9.0 (optimum pH 8.0). The biochemical test revealed that strain FM5 was positive for oxidase and catalase activities. It demonstrated the ability to decompose lysine and ornithine but not urea [30]. Additionally, strain FM5 utilized arabinose, cellobiose, melibiose, raffinose, glucose, and lactose as carbon sources but not affinose, xylose, inositol, or mannitol (Table 1). Phylogenetic analysis based on 31 housekeeping genes (e.g., *dnaG*, *ropB*, and *rplA*) indicated that strain FM5 shared 99.9% sequence similarity with *Priestia aryabhattai* (Figure 1), suggesting a close phylogenetic relationship with this species.

**Figure 2:**
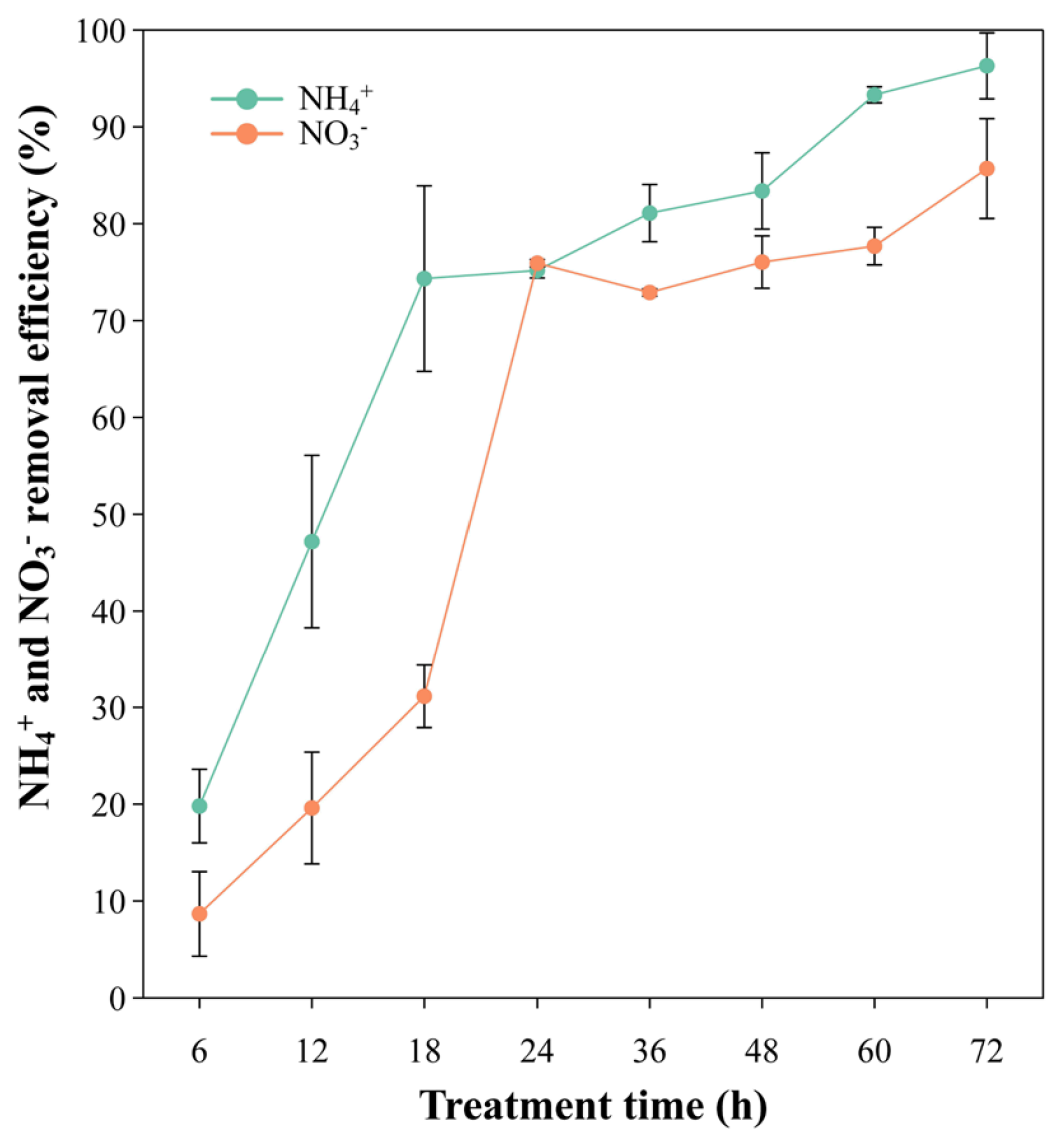
NH_4_^+^ and NO_3_^−^ removal capacity of strain FM5.

The NH_4_^+^ and NO_3_^−^ removal efficiency of strain FM5 for NH_4_^+^ and NO_3_^−^ was investigated in heterotrophic nitrification medium (HNM) and aerobic denitrification medium (ADM), respectively. Initial concentrations of NH_4_^+^ were set at 100 mg·L^−1^. After 24 h of incubation, both NH_4_^+^ and NO_3_^−^ concentrations decreased by 75% (Figure 2). By 72 h, NH_4_^+^ removal efficiency reached 95%, and NO_3_^−^ removal efficiency reached 85%.

### 3.2. NH_4_^+^ Removal Efficiency Under Different Cultivation Conditions

**Figure 3:**
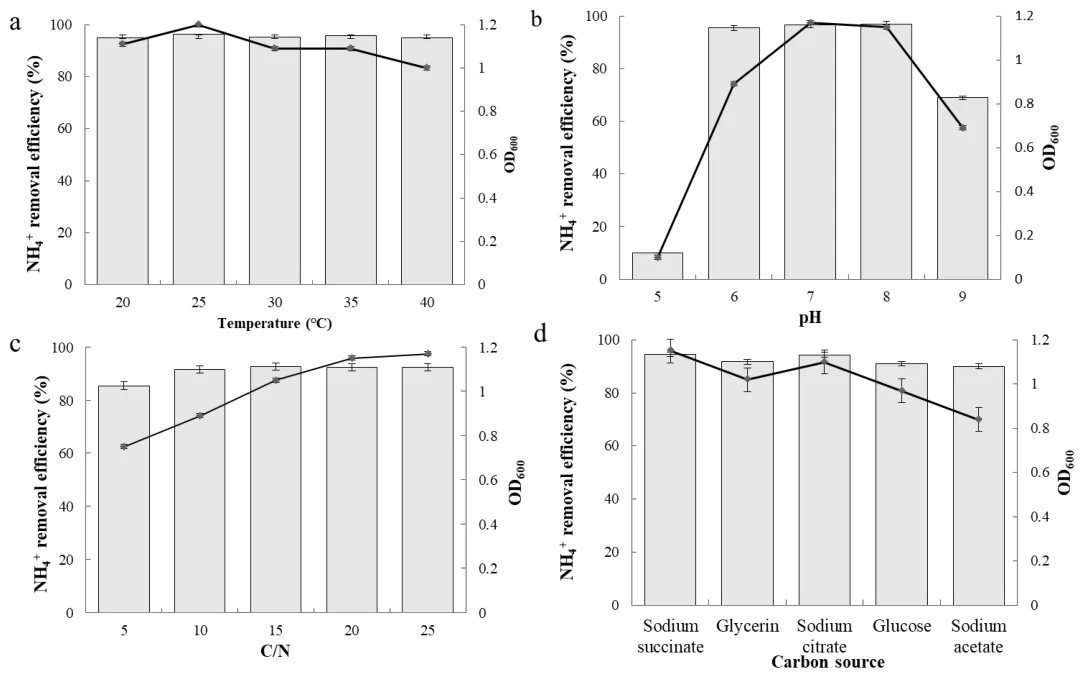
NH_4_^+^ removal characteristics of strain FM5 under different temperatures (**a**), pH (**b**), C/N (**c**), and carbon sources (**d**).

The NH_4_^+^ removal efficiency (%) of strain FM5 under different temperature, pH, C/N ratio, and carbon sources was investigated using single-factor experiments. The results showed that strain FM5 could efficiently remove NH_4_^+^ (94.8–96.3%), and the highest NH_4_^+^ removal efficiency (96.3%) was observed at 25 °C (Figure 3). The effect of pH on the NH_4_^+^ removal efficiency of FM5 demonstrated that the strain can effectively remove NH_4_^+^ within a pH range of 6.0–8.0. Strain FM5 achieved high NH_4_^+^ removal efficiency at C/N ratios of 15–25, with the highest efficiency observed at a C/N ratio of 20. Carbon source experiments demonstrated that strain FM5 effectively utilized sodium citrate and sodium succinate for NH_4_^+^ removal. In summary, the optimal conditions for NH_4_^+^ removal by strain FM5 were determined to be pH 8.0, temperature—25 °C, NH_4_^+^, and C/N ratio—20, with sodium succinate as the carbon source.

### 3.3. NO_3_^−^ Removal Efficiency Under Different Cultivation Conditions

**Figure 4:**
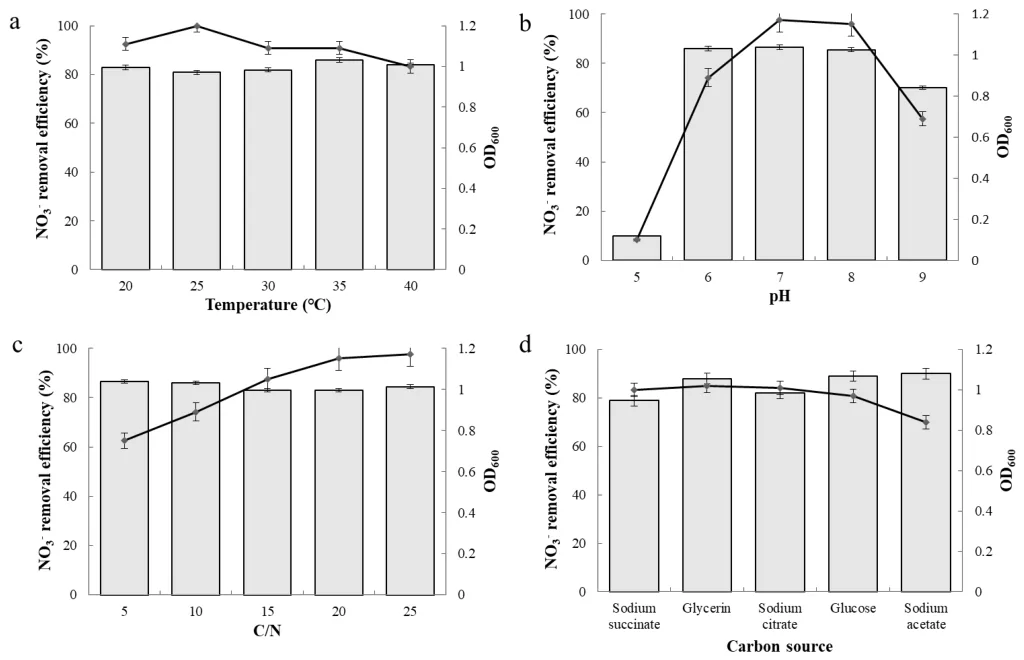
NO_3_^−^ removal characteristics of strain FM5 under different temperatures (**a**), pH (**b**), C/N (**c**), and carbon sources (**d**).

The results showed that strain FM5 could efficiently remove NO_3_^−^ (85.6%) at 35 °C (Figure 4), and a suitable temperature is essential for the growth of FM5. However, the highest OD_600_ was observed at 20 °C, rather than 35 °C. For the optimal pH, the highest NO_3_^−^ removal was observed at a pH value of 7.0, which was suitable for FM5 growth and enzyme activity. The suitable C/N ratio for NO_3_^−^ removal was 10, which was different from that for NH_4_^+^. Carbon source experiments demonstrated that strain FM5 effectively utilized sodium acetate for NO_3_^−^ removal.

### 3.4. Nitrogen Metabolism Pathway

**Figure 5:**
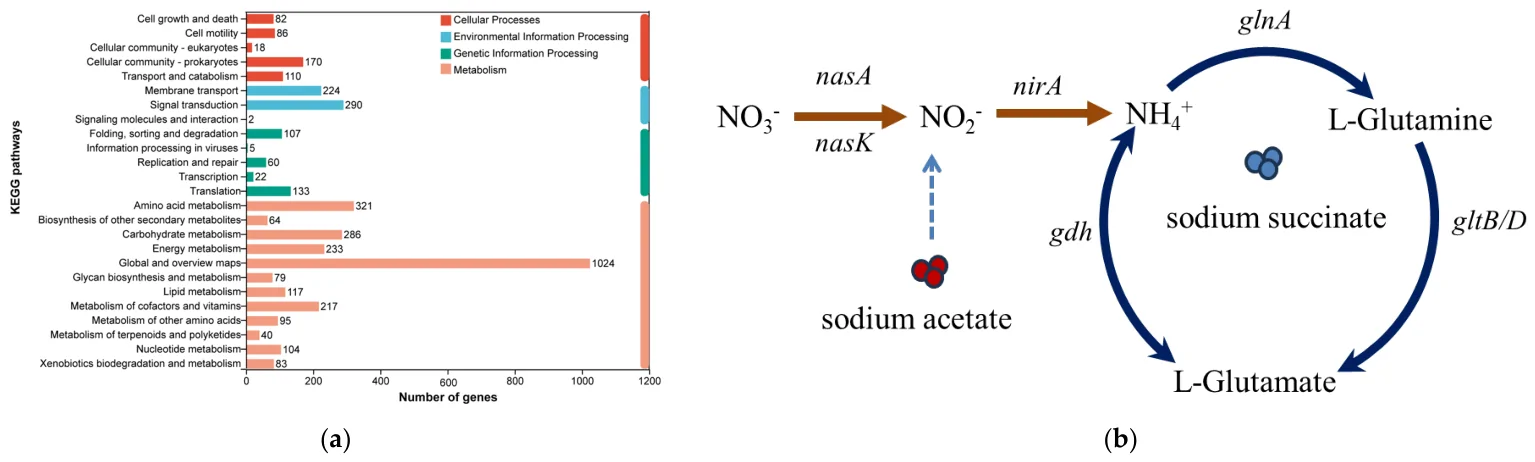
The KEGG profiles of FM5 (**a**) and the identified genes involved in NH_4_^+^ and NO_3_^−^ removal pathway (**b**). Red arrows: assimilatory nitrate reduction; blue arrows: glutamate metabolism.

The whole-genome data of strain FM5 has been submitted to GenBank (accession number: PRJNA1522652). The genomic data for FM5 had a GC content of 38.8%; the Kyoto Encyclopedia of Genes and Genomes (KEGG) results demonstrated that 2663 genes were involved in metabolism, including 233 genes in energy metabolism (Figure 5a). Annotation of the genome identified key genes involved in the nitrogen cycle, including assimilatory nitrate reductase (*nasA*), nitrite reductase (*nirA*), nitrate/nitrite transporters (*nrt*), glutamine synthase (*glnA*), glutamate synthase (*gltB/D*), and glutamate dehydrogenase (*gdhA*) (Figure 5b). These genes were functionally associated with assimilatory nitrate reduction and glutamate metabolism pathways.

### 3.5. NH_4_^+^ and COD Removal Capacity of Strain FM5 in Soymilk Wastewater

**Figure 6:**
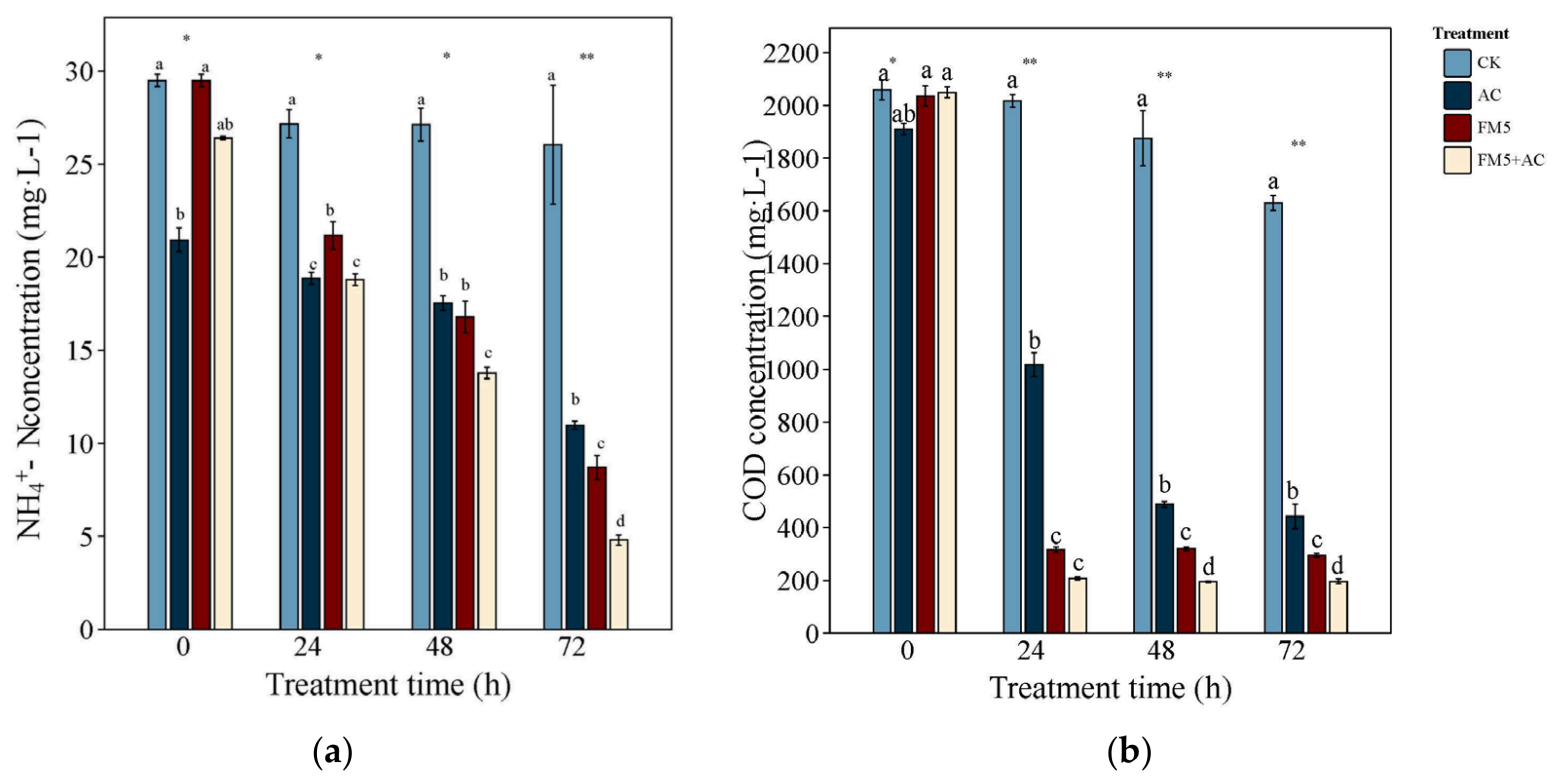
The NH_4_^+^-N (**a**) and COD (**b**) removal characteristics of strain FM5 in soymilk wastewater treatments (* *p* < 0.05, ** *p* < 0.01). Error bars denote standard deviation (SD).

Soy products are a major source of dietary protein, but their production generates significant volumes of wastewater, which poses environmental challenges without proper management [5]. In this study, the initial NH_4_^+^ concentration and COD in soymilk wastewater were measured as 29.5 mg·L^−1^ and 2058.3 mg·L^−1^. As shown in Figure 6a, the NH_4_^+^ concentrations in activated carbon (AC), FM5 and FM5 with AC decreased to 19.0, 21.2 and 18.8 mg·L^−1^ after 24 h of treatment and continued to decline steadily until 72 h (Figure 6). Finally, NH_4_^+^ concentrations were 11.0, 8.7 and 4.8 mg·L^−1^, representing, respectively, removal efficiencies of 62.7%, 70.4% and 83.7%. Furthermore, COD in the FM5 treatment decreased from 2058.3 mg·L^−1^ to 316.7 mg·L^−1^ at 24 h and 295.3 mg·L^−1^ at 72 h. In FM5 treatments with activated carbon, COD decreased to 196.0 mg·L^−1^ after 72 h of cultivation. The results demonstrated the effectiveness of FM5 and FM5 with AC in the treatment process.

## 4. Discussion

The NH_4_^+^ and NO_3_^−^ removal efficiency of strain FM5 reached 95% for NH_4_^+^ and 85% for NO_3_^−^ after 72 h in liquid media. At an initial NH_4_^+^ concentration of 120 mg·L^−1^, *Bacillus* sp. LY exhibited a removal efficiency of 73.7% [20]. Similarly, *Bacillus simplex* H-b achieved NH_4_^+^ and NO_3_^−1^ removal efficiencies of 82 and 67% in cold conditions, respectively [26].

Temperature, pH, C/N and carbon sources are confirmed as key parameters for NH_4_^+^ removal efficiencies [33,34]. Temperature is a critical factor influencing removal efficiencies, with an optimal temperature of 25–37 °C [33]. It was reported that a low pH value suppresses the growth and activity of most bacteria [34]. A previous study showed that bacteria generally prefer weak alkaline conditions for optimal growth and metabolism [10]. For instance, *Paracoccus denitrificans* strain HY-1 has demonstrated high NH_4_^+^ removal efficiency under weak alkaline conditions with pH ranges of 7.0 to 9.0 [33]. Carbon and nitrogen are essential for energy and electron donors for bacteria, and the initial C/N ratio significantly influences NH_4_^+^ removal efficiency [34]. For instance, with sodium succinate as the carbon source and a C/N ratio of 15, *Bacillus* sp. LY achieved a maximum NH_4_^+^ removal efficiency of 95% [20]. Similarly, *B. methylotrophicus* demonstrated high NH_4_^+^ removal efficiencies of 82% at a C/N ratio of 10 [26]. Similarly, *Paracoccus denitrificans* HY-1 was reported to efficiently remove NH_4_^+^ using sodium succinate as a carbon source [34]. *Bacillus* can utilize a wide range of organic carbon sources, including sodium citrate, sodium succinate, glucose, and acetate, for efficient NH_4_^+^ removal.

For NO_3_^−^ removal efficiency, the optimal temperature for nitrate removal (35 °C) was different from that for bacterial growth (20 °C). It is concluded that the optimal temperature (35 °C)—suitable for key enzymes in the denitrification process, including nitrate reductase and nitrite reductase—ranged between 30 and 40 °C [33]. For the optimal pH, a pH value of 7.0 was suitable for FM5 growth and enzyme activity. A suitable C/N ratio for NO_3_^−^ removal was observed at a value of 10. *Bacillus* strains exhibit optimal NO_3_^−^ removal performance at C/N ratios ranging from 10 to 20 [20,26]. It was also found that sodium acetate was beneficial for the growth of dissimilatory nitrate reduction to ammonia (DNRA) bacteria. It was concluded that acetate is preferred as an electron donor for the denitrification of high NO_3_^−^ concentrations [35].

In the genome data of strain FM5, genes involved in the nitrogen cycle, including the assimilatory nitrate reductase gene (*nasA*), the nitrite reductase gene (*nirA*), the nitrate/nitrite transporter gene (*nrt*), the glutamine synthetase gene (*glnA*), the glutamate synthase gene (*gltB/D*), and the glutamate dehydrogenase gene (*gdhA*), were annotated. In assimilatory nitrate reduction, nitrate was transported from extracellular nitrate into intracellular nitrate by *nrt*, which plays a critical role in nitrate/nitrite utilization. The transcriptional activity of *nrt* has been shown to increase under nitrogen-deficient conditions [36]. Within the nitrogen assimilation pathway, nitrate is first converted into nitrite by nitrate reductase (*nar*), followed by the conversion of nitrite into ammonia via nitrite reductase (*nir*) [33]. In strain FM5, nitrate is converted into nitrite by *nasA* and subsequently converted into ammonia by *nirA*. This transformation process is also reported in the heterotrophic nitrifying–aerobic denitrifying strain AHP123, where *nar* and *nir* catalyze the conversion of nitrate into ammonia [37]. Furthermore, ammonia can also participate in glutamate metabolism. It has been suggested that ammonia is assimilated by *Klebsiella* sp. through the glutamate dehydrogenase pathway [38]. Specifically, ammonia is first converted into glutamine by glutamine synthase (*glnA*) and then into glutamate by glutamate synthase (*gltB/D*). In the nitrogen assimilation pathway, nitrate is reduced to ammonia by nitrate reductase (*nar*) and nitrite reductase (*Nir*). Subsequently, ammonia is assimilated into glutamate via the actions of glutamine synthase (*glnA*), glutamate synthase (*gltB/D*), and glutamate dehydrogenase (*gdhA*) for cell growth [38,39,40]. Importantly, ammonia can also be converted into glutamate by *gdhA*. Importantly, ammonia can also be directly converted into glutamate by glutamate dehydrogenase (*gdhA*) [41,42]. *gdhA* catalyzes the reversible conversion between 2-oxoglutarate and glutamate, playing a crucial role in linking nitrogen and carbon metabolism [43]. The presence of these genes in the nitrogen assimilation pathway indicates that strain FM5 is capable of inorganic NH_4_^+^ and NO_3_^−^ removal.

Conventional treatment methods for soybean-processing wastewater include aerobic and anaerobic digestion [5]. However, the high operational costs associated with these methods often make direct disposal difficult, leading to substantial environmental pollution. *Priestia aryabhattai*, with biomineralization potential, was employed to effectively produce struvite from secondary-treated municipal wastewater [28]. In this study, *Priestia aryabhattai* FM5 was applied to treat soybean-processing wastewater. The results showed that strain FM5 was capable of removing 82.8% of NH_4_^+^-N in soybean-processing wastewater after 72 h of cultivation, with the addition of activated carbon. Activated carbon is one of the most commonly used adsorbents, with a high specific surface area, a well-developed porous structure, and a strong affinity for a wide range of contaminants [44]. Its versatility and effectiveness have made it a preferred material in wastewater treatment, particularly for the removal of organic pollutants, heavy metals, and emerging contaminants [45]. These results highlight the potential of NH_4_^+^ and NO_3_^−^ removal bacteria, such as strain FM5, in enhancing water quality and their applicability in environmental remediation. Further, a response surface methodology (RSM) is needed to assess interactive effects and optimize NH_4_^+^ and NO_3_^−^ removal by FM5.

## 5. Conclusions

The newly isolated *P. aryabhattai* FM5, with NH_4_^+^ and NO_3_^−^ removal capabilities, was applied for NH_4_^+^ removal in soymilk wastewater. The optimum conditions for NH_4_^+^ removal were pH 8.0, temperature—25 °C, and C/N ratio—20, with sodium succinate as the carbon source. The demonstration of *nasA*, *nirA*, *nrt*, *glnA*, *gltB/D* and *gdhA* suggested the NH_4_^+^ and NO_3_^−^ removal capability of *P. aryabhattai* FM5. Strain FM5 was capable of removing 4.40 mg·L^−l^ of NH_4_^+^ after 72 h of culturing, with the addition of activated carbon. Further research will focus on enhancing NH_4_^+^ and NO_3_^−^ removal capacity to promote the resource utilization of agricultural waste.

## Figures and Tables

**Table 1:** Biochemical tests for *P. aryabhattai* FM5.

Nitrogen Sources	Result	Carbon Sources	Result
Lysine	+	Arabinose	+
Ornithine	+	Cellobiose	+
Urea	+	Melibiose	+
		Raffinose	+
		Glucose	+
		Lactose	+
		Affinose	−
		Xylose	−
		Inositol	−
		Mannitol	−

## Data Availability

The original contributions presented in the study are included in the article. Further inquiries can be directed to the corresponding authors.

## References

[1] Qin P., Wang T., Luo Y. (2022). A review on plant-based proteins from soybean: Health benefits and soy product development. J. Agric. Food Res..

[2] King J., Leong S.Y., Alpos M., Johnson C., McLeod S., Peng M., Sutton K., Oey I. (2024). Role of food processing and incorporating legumes in food products to increase protein intake and enhance satiety. Trends Food Sci. Technol..

[3] Wu Y., Wang E., Gong W., Xu L., Zhao Z., He D., Yang F., Wang X., Yong T., Liu J. (2023). Soybean yield variations and the potential of intercropping to increase production in China. Field Crops Res..

[4] Paulinetti A.P., Augusto I.M.G., Batista L.P.P., Tavares A.G.B., Albanez R., Ratusznei S.M., Lovato G., Rodrigues J.A.D. (2022). Anaerobic digestion as a core process for sustainable energy production in the soybean biorefinery: A techno-economic assessment. Sustain. Horiz..

[5] Wang Y., Serventi L. (2019). Sustainability of dairy and soy processing: A review on wastewater recycling. J. Clean. Prod..

[6] Yu H.-Q., Hu Z.-H., Hong T.-Q., Gu G.-W. (2002). Performance of an anaerobic filter treating soybean processing wastewater with and without effluent recycle. Process Biochem..

[7] Liu N., Sun Z., Zhang H., Klausen L.H., Ryu M., Kang S. (2023). Emerging high-ammonia-nitrogen wastewater remediation by biological treatment and photocatalysis techniques. Sci. Total Environ..

[8] Bortoluzzi A.C., Demaman Oro C.E., dos Santos M.S.N., Mignoni M.L., Dallago R.M., Steffens J., Tres M.V. (2023). Combination of chemical coagulation and membrane-based separation for dairy wastewater treatment. J. Food Sci. Technol..

[9] Li J., Wan X., Wang H., Zhang Y., Ma Z., Yang W., Hu Y. (2024). Electrospun nanofibers electrostatically adsorb heterotrophic nitrifying and aerobic denitrifying bacteria to degrade nitrogen in wastewater. J. Environ. Manag..

[10] Xi H., Zhou X., Arslan M., Luo Z., Wei J., Wu Z., Gamal El-Din M. (2022). Heterotrophic nitrification and aerobic denitrification process: Promising but a long way to go in the wastewater treatment. Sci. Total Environ..

[11] Su M.-X., Cui Y.-W., Yang R.-C., Sui Y., Gu X.-Y., Li J.-Y., Lu Z.-Y. (2026). Static magnetic field coupled with control of sludge retention time enhancing nitrogen removal via heterotrophic nitrification-aerobic denitrification. J. Water Process Eng..

[12] Pan Z., Zhou J., Lin Z., Wang Y., Zhao P., Zhou J., Liu S., He X. (2020). Effects of COD/TN ratio on nitrogen removal efficiency, microbial community for high saline wastewater treatment based on heterotrophic nitrification-aerobic denitrification process. Bioresour. Technol..

[13] Yang L., Wang X.-H., Cui S., Ren Y.-X., Yu J., Chen N., Xiao Q., Guo L.-K., Wang R.-H. (2019). Simultaneous removal of nitrogen and phosphorous by heterotrophic nitrification-aerobic denitrification of a metal resistant bacterium *Pseudomonas putida* strain NP5. Bioresour. Technol..

[14] Yu J.-C., Wu Y.-J.-Z., Shin W.-S. (2025). From waste to value: Integrating legume byproducts into sustainable industrialization. Compr. Rev. Food Sci. Food Saf..

[15] Jo Y., Choi D., Shi X., Kim S.-H. (2026). Nitrogen removal performance, enrichment, and activity of *Anammox bacteria* in a backwashing free dynamic membrane bioreactor. J. Water Process Eng..

[16] Mao J., Ren M., Gong J., Qian Y. (2026). The influence of temperature and nitrogen source on ammonia removal and associated metabolism in activated sludge process for high-salinity nitrogen-containing wastewater treatment. J. Water Process Eng..

[17] Yang L., Ren Y.-X., Liang X., Zhao S.-Q., Wang J.-P., Xia Z.-H. (2015). Nitrogen removal characteristics of a heterotrophic nitrifier *Acinetobacter junii* YB and its potential application for the treatment of high-strength nitrogenous wastewater. Bioresour. Technol..

[18] Chen P., Li J., Li Q.X., Wang Y., Li S., Ren T., Wang L. (2012). Simultaneous heterotrophic nitrification and aerobic denitrification by bacterium *Rhodococcus* sp. CPZ24. Bioresour. Technol..

[19] Guo Y., Wang Y., Zhang Z., Huang F., Chen S. (2018). Physiological and transcriptomic insights into the cold adaptation mechanism of a novel heterotrophic nitrifying and aerobic denitrifying-like bacterium *Pseudomonas indoloxydans* YY-1. Int. Biodeterior. Biodegrad..

[20] Zhao B., He Y.L., Zhang X.F. (2010). Nitrogen removal capability through simultaneous heterotrophic nitrification and aerobic denitrification by *Bacillus* sp. LY. Environ. Technol..

[21] Yan P., Li T., Ren T., Zang Y., Sun S., Fan Y., Zhang Y., Gu X., He S. (2025). Heterotrophic denitrification enhancement via effective organic matter degradation driven by suitable iron dosage in sediment. J. Environ. Manag..

[22] Hu Y., Gu Y., Tan J., Ding C., Yu X., Li Z., Lin H. (2025). Effective denitrification from landfill leachate using magnetic PVA/CMC/DE carrier immobilized microorganisms. Waste Manag..

[23] Huang T.-Y., Ju H.-J., Huang M.-Y., Kuo Q.-M., Su W.-T. (2025). Optimal nitrite degradation by isolated *Bacillus subtilis* sp. N4 and applied for intensive aquaculture water quality management with immobilized strains. J. Environ. Manag..

[24] Li T., Zhan C., Guo G., Liu Z., Hao N., Ouyang P. (2021). Tofu processing wastewater as a low-cost substrate for high activity nattokinase production using *Bacillus subtilis*. BMC Biotechnol..

[25] Gomaa O.M., Ibrahim S.A.E.M., Mansour N.M. (2023). *Bacillus spizizenii* DN and microbial consortia biostimulation followed by gamma irradiation for efficient textile wastewater treatment. Environ. Sci. Pollut. Res..

[26] Yang Q., Yang T., Shi Y., Xin Y., Zhang L., Gu Z., Li Y., Ding Z., Shi G. (2021). The nitrogen removal characterization of a cold-adapted bacterium: *Bacillus simplex* H-b. Bioresour. Technol..

[27] Sharma K., Sharma S., Vaishnav A., Jain R., Singh D., Singh H.B., Goel A., Singh S. (2022). Salt-tolerant PGPR strain *Priestia endophytica* SK1 promotes fenugreek growth under salt stress by inducing nitrogen assimilation and secondary metabolites. J. Appl. Microbiol..

[28] Nithyashree J.K., Subramanian S. (2025). Biomineralization Potential of *Priestia aryabhattai*, a Freshwater Isolate for Phosphorus Recovery through Struvite Production from Municipal Wastewater. Geomicrobiol. J..

[29] Zhu L., Ding W., Feng L.-J., Kong Y., Xu J., Xu X.-Y. (2012). Isolation of aerobic denitrifiers and characterization for their potential application in the bioremediation of oligotrophic ecosystem. Bioresour. Technol..

[30] Chen Q., Liu B., Wang J., Che J., Liu G., Guan X. (2017). Antifungal Lipopeptides Produced by *Bacillus* sp. FJAT-14262 Isolated from Rhizosphere Soil of the Medicinal Plant *Anoectochilus roxburghii*. Appl. Biochem. Biotechnol..

[31] Chen S. (2023). Ultrafast one-pass FASTQ data preprocessing, quality control, and deduplication using fastp. iMeta.

[32] Yu H., Ma C., Quan X., Chen S., Zhao H. (2009). Flow Injection Analysis of Chemical Oxygen Demand (COD) by Using a Boron-Doped Diamond (BDD) Electrode. Environ. Sci. Technol..

[33] Song T., Zhang X., Li J., Wu X., Feng H., Dong W. (2021). A review of research progress of heterotrophic nitrification and aerobic denitrification microorganisms (HNADMs). Sci. Total Environ..

[34] Huan C., Yan Z., Sun J., Liu Y., Zeng Y., Qin W., Cheng Y., Tian X., Tan Z., Lyu Q. (2022). Nitrogen removal characteristics of efficient heterotrophic nitrification-aerobic denitrification bacterium and application in biological deodorization. Bioresour. Technol..

[35] Nie W.-B., Ding J., Xie G.-J., Tan X., Lu Y., Peng L., Liu B.-F., Xing D.-F., Yuan Z., Ren N. (2021). Simultaneous nitrate and sulfate dependent anaerobic oxidation of methane linking carbon, nitrogen and sulfur cycles. Water Res..

[36] Li H., Li L., Yu L., Yang X., Shi X., Wang J., Li J., Lin S. (2021). Transcriptome profiling reveals versatile dissolved organic nitrogen utilization, mixotrophy, and N conservation in the dinoflagellate *Prorocentrum shikokuense* under N deficiency. Sci. Total Environ..

[37] Zhou X., Wang Y., Tan X., Sheng Y., Li Y., Zhang Q., Xu J., Shi Z. (2023). Genomics and nitrogen metabolic characteristics of a novel heterotrophic nitrifying-aerobic denitrifying bacterium *Acinetobacter oleivorans* AHP123. Bioresour. Technol..

[38] Jin P., Chen Y., Yao R., Zheng Z., Du Q. (2019). New insight into the nitrogen metabolism of simultaneous heterotrophic nitrification-aerobic denitrification bacterium in mRNA expression. J. Hazard. Mater..

[39] Pal R.R., Khardenavis A.A., Purohit H.J. (2015). Identification and monitoring of nitrification and denitrification genes in *Klebsiella pneumoniae* EGD-HP19-C for its ability to perform heterotrophic nitrification and aerobic denitrification. Funct. Integr. Genom..

[40] Zhang Y., Li B., Najman M.A., Xu C., Huang S., Kim D.-H., Kim S.H., Kang S., Liu C., Ratnaweera H. (2026). Evaluation of nitrogen removal characteristics of *Bacillus spizizenii* HH1-2 by nitrogen assimilation: Isolation, performance and genomics. Bioresour. Technol..

[41] Ge F., Sun J., Ren Y., He B., Li J., Yang S., Li W. (2022). Transcriptomic and enzymatic analysis reveals the roles of glutamate dehydrogenase in *Corynebacterium glutamicum*. AMB Express.

[42] Dong Y., Wang Z., Li L., Zhang X., Chen F., He J. (2023). Heterotrophic nitrification and aerobic denitrification characteristics of the psychrotolerant *Pseudomonas peli* NR-5 at low temperatures. Bioprocess Biosyst. Eng..

[43] Kang X., Zhao X., Song X., Wang D., Shi G., Duan X., Chen X., Shen G. (2023). Nitrogen removal by a novel strain *Priestia aryabhattai* KX-3 from East Antarctica under alkaline pH and low-temperature conditions. Process Biochem..

[44] Devi R., Kumar V., Kumar S., Bulla M., Jatrana A., Rani R., Mishra A.K., Singh P. (2023). Recent advancement in biomass-derived activated carbon for waste water treatment, energy storage, and gas purification: A review. J. Mater. Sci..

[45] Barczak B., Januszewicz K. (2025). Advancing sustainable wastewater treatment with biomass-based highly porous activated carbon: Insights into sorption mechanisms and efficiency. J. Environ. Chem. Eng..

